# It’s In The Bag: Tidal Volumes in Adult and Pediatric Bag Valve Masks

**DOI:** 10.5811/westjem.2020.3.45788

**Published:** 2020-04-27

**Authors:** Benjamin Dafilou, Daniel Schwester, Nathan Ruhl, Andreia Marques-Baptista

**Affiliations:** * Capital Health Hospital System, Department of Emergency Medical Services, Trenton, New Jersey; † Rowan University, Department of Biological Sciences, Glassboro, New Jersey

## Abstract

**Introduction:**

A bag valve mask (BVM) is a life saving device used by all levels of health care professionals during resuscitative care. We focus most of our time optimizing the patient’s position, firmly securing the mask, and frequency of ventilations. However, despite our best efforts to control these factors, we may still be precipitating harm to the patient. Multiple studies have shown the tidal volumes typically delivered by the adult BVM are often higher than recommended for lung-protective ventilation protocols. In this study we measure and compare the ventilation parameters delivered by the adult and pediatric BVM ventilators.

**Methods:**

A RespiTrainer Advance® adult mannequin was used to simulate a patient. Healthcare providers were directed to manually ventilate an intubated mannequin for two minutes using adult and pediatric sized BVMs. Tidal volume, minute ventilation, peak pressure, and respiration rate was recorded.

**Results:**

The adult BVM provided a mean tidal volume of 807.7mL versus the pediatric BVM providing 630.7mL, both of which exceeded the upper threshold of 560mL of tidal volume necessary for lung protective ventilation of an adult male with an ideal body weight of 70kg. The adult BVM exceeded this threshold by 44.2% versus the pediatric BVM’s 12.6% with 93% of participants exceeding the maximum threshold with the adult BVM and 82.3% exceeding it with the pediatric BVM.

**Conclusion:**

The pediatric BVM in our study provided far more consistent and appropriate ventilation parameters for adult patients compared to an adult BVM, but still exceeded the upper limits of lung protective ventilation parameters. The results of this study highlight the potential dangers in using an adult BVM due to increased risk of pulmonary barotrauma. These higher tidal volumes can contribute to lung injury. This study confirms that smaller BVMs may provide safer ventilatory parameters. Future studies should focus on patient-centered outcomes with BVM.

## INTRODUCTION

High volumes delivered during positive pressure ventilation can precipitate lung injury in a patient already suffering from an underlying pulmonary pathology. Barotrauma refers to damage sustained to the lung from rapid or excessive increases in pressure. Volutrauma describes structural lung injury due to over-distention of the alveoli that occurs when higher than physiologic volumes are delivered. Barotrauma is defined as trauma caused by rapid or extreme changes in pressure affecting enclosed cavities within the body.^1^ Positive pressure ventilation provided via bag valve masks (BVMs) may expose patients to high airway pressures and volumes, potentiating similar alveolar damage. Conditions such as interstitial emphysema, pneumothorax, pneumomediastinum, subcutaneous emphysema, and pneumoperitoneum are clinical presentations of barotrauma.^2^ The purpose of the study is to determine whether healthcare providers are unintentionally delivering pressures and volumes that could potentiate injury during manual ventilation using BVMs.

Stroke volumes of BVMs are defined by the manufacturer as the projected delivered tidal volume by manually squeezing the bag. To achieve lung-protective ventilation for intubated patients, the average tidal volume should be between 5–8 milliliters per kilogram (mL/kg) of ideal body weight.^3^,^4^,^5^,^6^ The reservoirs of adult BVMs contain between 1500–2000 mL of air, depending on manufacturer and model, with projected stroke volumes of between 900–1000 mL.^7^,^8^,^9^ The volume of pediatric BVMs can range anywhere between 500–1000 mL with stroke volumes of 450–650 mL,^7^,^8^,^9^ closer to the targeted tidal volume for adult patients who are critically ill or in cardiac arrest.^3^ We assessed adult and pediatric BVM ventilation in a simulated scenario, comparing the mean tidal volume, peak pressure, and respiratory rate for each.

## METHODS

### Study Setting

This study took place at Capital Health Hopewell Medical Center, Capital Health Regional Medical Center, and the 2016 New Jersey Statewide Conference on Emergency Medical Services (EMS). One hundred and thirty people participated in this study: 1 patient care advocate, 1 licensed practical nurse, 4 respiratory therapists, 5 physician assistants, 11 critical care technicians, 13 medical doctors, 25 paramedics, 28 emergency medical technicians, and 42 registered nurses. All participants are active health care providers working in the in-hospital or pre-hospital setting. All data was collected between September and October of 2016. Participants were selected out of convenience and those willing to participate.

### Study Design

Institutional Review Board approval was given for this study. This study was conducted using the QuickLung RespiTrainer Advance® set to the adult setting, which means that the respiratory mechanics were set to a compliance of 50 milliliters per centimeter of water (mL/cm H_2_O) and a resistance of 5 centimeters of water per liter per second (cmH_2_O/L/s). These settings allowed for the RespiTrainer® to accurately calculate tidal volumes (V_t_), peak pressures (P_peak_), breath rates (BR), and minute ventilations (MV). P_peak_ was recorded by the RespiTrainer Advance as the highest value of pressure during a single positive pressure ventilation. MV is calculated by the RespiTrainer Advance as the prorated average tidal volume per minute from a sample of one breath. BR were calculated by the RespiTrainer Advance in real time from the previous breath and reported as the average of these measurements. V_t_ were calculated by V_t_ = (P_peak_ − P_min_) / (50 mL/cm H_2_O)

The RespiTrainer® was intubated with a standard size 7.5 millimeters (mm) endotracheal tube at 25 centimeters (cm) at the lip. The endotracheal cuff was then inflated with 10 mL of air. The chest rise mechanism was not utilized during data collection because, during a real cardiac arrest, clinicians providing ventilations would not be able to see chest rise while compressions were in progress in an intubated patient. An AirFlow AF1140MB Adult BVM® and an AirFlow AF2140MB Pediatric BVM® were used for this study. The range of tidal volumes used for this study for an adult male patient with an ideal body weight of 70 kg was 350–560 mL based off a lung protective range of 5–8 mL/kg.^10^ The adult BVM, an AirFlow AF1140MB, had a maximum capacity of 1900mL and the pediatric BVM, an AirFlow AF2140MB, had a maximum capacity of 1000mL.

**Population Health Research Capsule**

What do we already know about this issue?

Healthcare providers at all levels are generally very ineffective at providing appropriate ventilations with bag valve masks.

What was the research question?

Whether bag valve masks (BVM) provide appropriate tidal volume for lung protective ventilation.

What was the major finding of the study?

The tidal volumes provided by standard size BVMs significantly exceed safe thresholds for lung protective ventilation.

How does this improve population health?

BVMs are used widely to resuscitate and ventilate critically ill patients, and they may actually be causing harm in practical use.

A simulated cardiac arrest scenario was selected to encourage providers to ventilate slowly and use lower volumes. This standardized approach allowed observation of the true ventilatory metrics delivered when using the two BVMs. Prior to data collection, each participant was given the following instruction: “You are in a cardiac arrest scenario. You have been directed to provide ventilations to an adult intubated patient for two minutes of cardio-pulmonary resuscitation (CPR) using an adult BVM; and then another two minutes, using a pediatric BVM.” Each participant was instructed that they were only responsible for ventilations; they did not need to provide compressions, medications, pause for pulse checks or any other CPR related activity. The only demographic information collected for the participants was their highest medical certification level.

### Statistical Analysis

All data was analyzed using JMP 12.0. Sample size was not sufficient to test for interactive effects between the different metrics of BVM performance (tidal volume, peak pressure, respiration rate and minute ventilation) and the different certification types of study participants, so differences in adult vs. pediatric BVM performance were analyzed using discrete Wilcoxon signed-rank tests (paired differences). Wilcoxon signed-rank tests were also used to compare tidal volume for both adult and pediatric BVMs to an idealized upper-threshold of 560 mL (upper threshold for an adult male with an ideal body weight of 70 kg).^3^

## RESULTS

The four metrics measured during this study were tidal volume in mL, respiratory rate in breaths per minute (bpm), peak pressure in cmH_2_O, and minute ventilation in liters (L). There was a significant difference between adult and pediatric BVM performance (Table 1) as measured by tidal volume (p=<0.001), peak pressure (p=<0.001), and minute ventilation (p=<0.001), but not respiration rate (p=0.549).

The mean tidal volume measured using the adult BVM was 807.7 mL versus the pediatric BVM mean tidal volume of 630.7 mL. The mean peak pressure measured in the adult BVM was 17 cmH_2_O versus the mean peak pressure of the pediatric BVM of 13.4 cmH_2_O. The mean minute ventilation measured for the adult BVM was 11.6 L versus 8.8 L for the pediatric BVM. The mean respiration rate measured with the adult BVM was 14.2 bpm versus 13.9 bpm in the pediatric BVM group.

**Figure 1:**
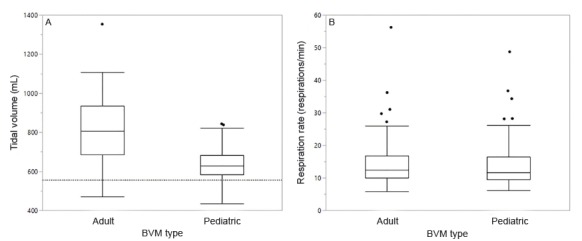
Side-by-side boxplots of adult vs. pediatric for mean tidal volume (A) and mean respiration rate (B). The dashed line in A represents the idealized upper threshold for an adult male with an ideal body weight of 70 kilograms (kg) at 8 milliliters (mL)/kg, or 560 mL. *BVM*, bag valve mask; *min*, minutes.

Tidal volume for both adult (p=<0.001) and pediatric (p=<0.001) BVMs significantly exceeded the threshold of 560 mL for an adult male with an ideal body weight of 70 kg, but the difference was far greater for the adult BVM (Figure 1A; adult mean tidal volume = 807.7 mL; pediatric mean tidal volume = 630.7 mL). The mean tidal volume delivered by the adult BVM exceeded the upper threshold of 560 mL for an adult male with an ideal body weight of 70 kg patient by 44.2%, versus the pediatric BVM where the mean tidal volume exceeded the upper threshold by 12.6%. The mean measured peak pressure for the adult BVM was 26.9% higher than it was in the pediatric BVM. The mean measured minute ventilation for the adult BVM was 31.8% higher than it was in the pediatric BVM.

While both BVMs are capable of delivering appropriate tidal volumes, 93% (n=121) of participants exceeded the upper threshold for tidal volumes using the adult BVM and 82.3% (n=107) exceeded the upper threshold for tidal volumes using the pediatric BVM.

## DISCUSSION

Studies have shown that ventilation using low tidal volumes is associated with reduced morbidity and mortality.^4^,^5^,^6^,^11^,^12^ Higher tidal volumes can lead to increased organ dysfunction and inflammation in intubated patients.^5^,^6^ Ideal conditions for intubated patients on mechanical ventilation is a tidal volume of 5–8 mL/kg, or 350–560 mL in an adult male with an ideal body weight of 70 kg.^10^ Most providers in our study ventilated the simulator mannequin with over 800 mL of tidal volume using the adult BVM (Figure 1A and Table 1), which is over 200 mL higher than the upper threshold of most recommended lung-protective ventilator settings.^13^ The pediatric BVM provided slightly elevated, but more physiologically appropriate, tidal volumes and peak pressures for adult patients. Although our study was not conducted on patients, exceeding the physiologically appropriate metrics could have a negative impact on patient care due to the consequences of barotrauma and volutrauma. Studies have consistently shown low volume mechanical ventilation in the setting of acute lung injury results in significantly lower mortality.^11^,^12^

There was no significant difference in breaths per minute when using the BVMs. This is significant because even in a simulated environment under ideal conditions all providers consistently ventilated above the recommended rate 8–10 bpm in a cardiac arrest scenario.^14^ Ventilating at higher than recommended rates potentiates the damage caused by the higher volumes and pressures. As shown in Figure 1B, there was no significant difference in respiratory rate between the adult and pediatric BVMs, which indicates that participants understood the directions correctly and did not switch ventilatory rates when switching BVMs.

This study adds to an emerging body of literature on the use of smaller BVMs^5^ for achieving closer to ideal physiologic parameters during manual ventilations of intubated patients.^4^,^5^,^6^ Siegler et al examined whether or not pediatric BVMs could provide sufficient tidal volume to adult patients via several different airway securing devices. Though they had a smaller cohort, their results were similar to our own.

## LIMITATIONS

This study is limited to the accuracy of the Quick Lung RespiTrainer Advance®. It is a very advanced simulator, but it assumes standard pulmonary compliance and resistance whereas human subjects vary widely and significantly. This study was conducted under a controlled environment that differs from a true patient care situation.^15^ We did not randomize the order in which we conducted ventilations with the different BVM types, so it is possible that some amount of variation between BVM types could be due to factors such as fatigue, but the observation that respiratory rate did not decline between treatments (Figure 1B) indicates that fatigue was not a meaningful issue in this study. We were also limited by the fact that this study does not include human patients and therefore could not measure patient outcomes or complications. The fact that the adult BVM was always used first may have influenced subjects to provide more volume with the pediatric BVM because the order was not randomized. Also, the lack of chest wall movement because this was a simulated cardiac arrest scenario may have caused subjects to provide more volume than they normally would if chest compressions were not being performed. Additionally, although we were simulating a cardiac arrest scenario because we did not use the chest rise function of the mannequin participants may have overventilated the mannequin due to not being able to see chest rise. This study was also limited because it only did an analysis for an adult male patient with an ideal body weight of 70 kg, this is significantly higher than a female adult patient.

## CONCLUSION

The results of this study showed extreme tidal volumes were delivered while using a standard size adult BVM. The pediatric BVM in our study provided far more consistent and appropriate ventilation compared to an adult BVM in a simulated adult patient, though it still exceeded upper limits for lung-protective ventilation. Additional data obtained from clinical trials comparing a smaller or newly designed BVM to standard BVM are needed; however, it seems prudent to consider reducing the size or redesigning the standard adult BVM to minimize the risk of barotrauma.

## Figures and Tables

**Table 1:** Mean and standard deviation (SD) for adult and pediatric bag valve mask metrics and results from Wilcoxon Signed-Rank analysis.

	Adult		Pediatric		Wilcoxon Signed-Rank Test		
			
BVM metric	Mean	SD	Mean	SD	Difference	SD	P value
			
Tidal volume (mL)	807.7	160.3	630.7	84.9	177	111.9	<0.001
Respiration rate (RR)	14.2	6.7	13.9	6.6	0.3	3.2	0.549
Peak pressure (cm H_2_O)	17	3.8	13.4	2.4	3.6	2.3	<0.001
Minute ventilation (L)	11.6	6.1	8.8	4.7	2.7	2.9	<0.001

*BVM*, bag valve mask; *mL*, milliliters; *RR*, respirations per minute; *cm H_2_O*, centimeters of water; *L*, liters.

Differences were calculated as Adult-Pediatric; positive values indicate adult metrics were higher.

## References

[1] WCS Barotrauma. MedicineNet.

[2] Naval Sea US, Command Systems (2006). US Navy Diving Manual.

[3] Siegler J, Kroll M, Wojcik S (2016). Can EMS Providers Provide Appropriate Tidal Volumes in a Simulated Adult-sized Patient with a Pediatric-sized Bag-Valve-Mask?. Prehosp Emerg Care.

[4] Gu W-J, Wang F, Liu J-C (2015). Effect of lung-protective ventilation with lower tidal volumes on clinical outcomes among patients undergoing surgery: a meta-analysis of randomized controlled trials. CMAJ.

[5] Lellouche F, Dionne S, Simard S (2012). High Tidal Volumes in Mechanically Ventilated Patients Increase Organ Dysfunction after Cardiac Surgery. Anesthesiology.

[6] Oliveira RPD, Hetzel M, Silva MDA (2010). Mechanical ventilation with high tidal volume induces inflammation in patients without lung disease. Crit Care.

[7] Ventlab (2017). Airflow Manual Resuscitator. Airflow Manual Resuscitator.

[8] Ambu (2017). Ambu Spur II. Ambu Spur II.

[9] Intersurgical (2017). Bag-Valve-Mask (BVM) manual resuscitation systems. Bag-Valve-Mask (BVM) manual resuscitation systems.

[10] Kilickaya O, Gajic O (2013). Initial ventilator settings for critically ill patients. Crit Care.

[11] (2000). Ventilation with Lower Tidal Volumes as Compared with Traditional Tidal Volumes for Acute Lung Injury and the Acute Respiratory Distress Syndrome. N Engl J Med.

[12] Amato MBP, Barbas CSV, Medeiros DM (1998). Effect of a Protective-Ventilation Strategy on Mortality in the Acute Respiratory Distress Syndrome. N Engl J Med.

[13] Brower RG, Matthay MA, Morris A (2000). Ventilation with Lower Tidal Volumes as Compared with Traditional Tidal Volumes for Acute Lung Injury and the Acute Respiratory Distress Syndrome. N Engl J Med.

[14] Link MS, Berkow LC, Kudenchuk PJ (2015). Part 7: Adult Advanced Cardiovascular Life Support. Circulation.

[15] Elling R, Politis J (1983). An evaluation of emergency medical technicians ability to use manual ventilation devices. Ann Emerg Med.

